# Feasibility of robotic cholecystectomy at an academic center with a young robotic surgery program: a retrospective cohort study with umbrella review

**DOI:** 10.1007/s11701-024-01824-x

**Published:** 2024-02-27

**Authors:** Maria Paula Corzo, Daniel Tomey, Alessandro Martinino, Roberto Secchi, Steven Elzein, Yoon Kyung Lee, Adel Abou-Mrad, Rodolfo J. Oviedo

**Affiliations:** 1 Facultad de Medicina, Universidad de Los Andes, Bogota, Colombia; 2 Department of Surgery, Houston Methodist Hospital, Houston, TX USA; 3 Department of Surgery, University of Illinois at Chicago, Chicago, IL USA; 4 Département de Chirurgie, Centre Hospitalier Régional d’Orléans, Orléans, France; 5 Nacogdoches Center for Metabolic & Weight Loss Surgery, Nacogdoches, TX USA; 6 University of Houston Tilman J. Fertitta Family College of Medicine, Houston, TX USA; 7 Sam Houston State University College of Osteopathic Medicine, Conroe, TX USA

**Keywords:** Cholecystectomy, Minimally invasive surgery, Robotic, Laparoscopic

## Abstract

Laparoscopic cholecystectomy (LC) has been standard of care for surgical treatment of benign gallbladder pathology for decades. With the advent of robotic surgical technology, robotic cholecystectomy (RC) has gained attention as an alternative to conventional laparoscopy. This study introduces a single-surgeon experience with laparoscopic versus robotic cholecystectomy and an umbrella systematic review of the outcomes of both approaches. A retrospective chart review was performed at a single institution on a prospectively maintained database of patients undergoing laparoscopic or robotic cholecystectomy for benign gallbladder pathology. An umbrella systematic review was conducted using PRISMA methodology. A total of 103 patients were identified; 61 patients underwent LC and 42 underwent RC. In the RC cohort, 17 cases were completed using a four-port technique while 25 were completed using a three-port technique. Patients undergoing RC were older compared to the LC group (44.78 vs 57.02 years old; *p* < 0.001) and exhibited lower body mass index (29.37 vs 32.37 kg/m^2^, *p* = 0.040). No statistically significant difference in operative time or need for postoperative ERCP was noted. Neither this series nor the umbrella systematic review revealed significant differences in conversion to open surgery or readmissions between the LC and RC cohorts. Three-port RC was associated with reduced operative time compared to four-port RC (101.28 vs 150.76 min; *p* < 0.001). Robotic cholecystectomy is feasible and safe at a young robotic surgery program in an academic center setting and comparable to laparoscopic cholecystectomy clinical outcomes.

**Supplementary Information:**

The online version contains supplementary material available at 10.1007/s11701-024-01824-x.

## Introduction

Surgery can be associated with significant morbidity, pain, prolonged hospitalization, and even mortality. To reduce these risks, surgeons have developed and are trained to perform minimally invasive surgery, including laparoscopic and robotic approaches. Robotic surgery is an advanced extension of traditional laparoscopy that incorporates sophisticated tools to increase the ergonomic advantage for surgeons, including: endoscope control with three-dimensional visualization of the surgical field, enhanced articulation of instruments, and control of multiple arms [1]. This allows many surgeons to perform procedures with increased finesse and versatility compared to their abilities in conventional laparoscopy.

Despite these advantages, robotic surgery has been associated with increased cost and longer operating times [2, 3]. The current literature reports contradicting results regarding the effect of robotic surgery on patient outcomes. *Walker *et al. reported that compared to a laparoscopic approach, robotic ventral hernia repair was associated with decreased incidence of hernia recurrence and surgical site occurrence such as surgical site infection and seroma formation [4]. Furthermore, *Waite *et al. reported that robotic inguinal hernia repair decreased time spent in the recovery room and improved patient-reported pain scores compared to laparoscopic repair, despite an increase in operative time [5].

The goal of this study is to explore the differences in clinical outcomes of patients undergoing a minimally invasive cholecystectomy with the laparoscopic and the robotic approaches at a single academic center with a young robotic surgery program. In addition, an umbrella systematic review was conducted to compare the results from this single center, single surgeon experience to the reported literature. The results generated from this and other similar studies may help to reduce practice variability in our healthcare system and improve the overall patient and surgeon experience in minimally invasive cholecystectomy.

## Methods

A single-center retrospective chart review was performed to identify patients who underwent laparoscopic cholecystectomy (LC) or robotic cholecystectomy (RC) between November 2020 and January 2022 at an urban academic medical center with a recently established robotic surgery program. Operations were performed by a single board-certified minimally invasive surgeon with significant experience and case numbers in the thousands for both techniques. The data were collected from a prospectively maintained database within the institution’s electronic health record system in accordance with Institutional Review Board regulations (Protocol #PRO00031398). Patients over the age of 18 who underwent a minimally invasive cholecystectomy were included in the study. Patients under the age of 18 or those who underwent an open cholecystectomy from the beginning (not a decision to convert to open) were excluded. There were no other inclusion or exclusion criteria for this study.

The primary endpoint for the analyses was 30-day morbidity. Secondary endpoints included operative time in minutes, estimated blood loss, conversion to open, intensive care unit (ICU) admission, length of stay in hours, incidence of bile leak, abscess, surgical site infection, post-operative small bowel obstruction (SBO), post-operative emergency department (ED) visit, post-operative endoscopic retrograde cholangiopancreatography (ERCP), blood transfusion within 30 days, and 30-day readmission. Additional variables collected included age, sex, body mass index (BMI), and American Society of Anesthesiologists (ASA) physical status class. Patient characteristics were reported as frequencies and proportions for categorical variables and as mean values for continuous variables. Differences between groups (laparoscopic cholecystectomy versus robotic cholecystectomy) were determined by paired t-tests for categorical variables. A *p*-value less than 0.05 was considered statistically significant. All analyses were performed on the SPSS Statistics software (Version 27, IBM). This study was approved by the Houston Methodist Research Institute’s Institutional Review Board.

LC was performed in the standard fashion with four ports, with optical entry for access into the peritoneal cavity, anterolateral retraction at the infundibulum, cephalad retraction at the fundus, and dissection of the structures comprising the critical view of safety first as opposed to a top–down approach. RC was performed both with the da Vinci Xi robotic platform (Sunnyvale, CA, USA) in the same manner with either three or four surgical ports arranged transversely and infra-umbilically with the adjunct of indocyanine green (ICG) fluorescence in all cases to assist with identification of the structures comprising the critical view of safety. Selection criteria for the 3-port technique were the need for cholecystectomy and availability of the robotic platform. If the robotic platform was available, the first choice was to perform the cholecystectomy with 3 ports only, unless there was a significant amount of inflammation from the beginning or noticed throughout the case which in the surgeon’s judgement dictated the need for all four arms of the robotic to be used. In such cases, the 4-port technique was adopted and implemented out of safety to facilitate retraction and dissection, although it was not the first choice. ICG was used only for RC at our institution. While the use of ICG in all cholecystectomies is ideal, ICG was noted only for RC in this analysis due to inconsistent availability of a laparoscopic tower and equipment compatible with ICG fluorescence. Otherwise, all the procedures should have been done with ICG based on its value and contributions to patient safety. An intraoperative cholangiogram was conducted selectively based on clinical and imaging criteria only for both the LC and the RC cohorts.

## Results

A total of 103 patients who underwent minimally invasive cholecystectomy by a single surgeon with resident participation during the critical steps or all of the steps of the operation at an academic medical center were identified. Of these patients, 61 underwent LC and 42 underwent RC. In the RC group, 17 cases were completed using four surgical ports, and 25 cases were completed using three ports. A total of 61 of the surgical cases were done emergently, whereby 48 were LC and 13 were RC. A summary of the differences between patients undergoing LC and RC is provided in Table 1. Patients undergoing RC were older (57.02 vs 44.78 years old, *p* < 0.001) and exhibited a lower BMI (29.37 vs 32.37 kg/m^2^, *p* = 0.040) than those undergoing LC. A higher proportion of patients undergoing LC did so emergently whereas a higher proportion of RC cases were planned.

**Table 1:** Outcomes of laparoscopic and robotic cholecystectomy

	Laparoscopic (*N* = 61)	Robotic assisted (*N* = 42)	*P*-value
Age, mean (± SD)	44.78 (19–87)	57.02 (27–83)	** < 0.001**
Sex, *n* (%)			0.909
Female	37 (60.66%)	25 (59.52%)	
Male	24 (39.34%)	17 (40.48%)	
BMI, mean (± SD)	32.37 (20.36–60.33)	29.38 (19.66–49.26)	**0.040**
ASA, *n* (%)			0.089
I	3 (4.92%)	1 (2.38%)	
II	37 (60.65%)	19 (45.24%)	
III	18 (29.51%)	19 (45.24%)	
IV	3 (4.92%)	3 (7.14%)	
Type of cholecystectomy			** < 0.001**
Emergent	48 (78.68%)	13 (30.95%)	
Planned	13 (21.32%)	29 (69.04%)	
Conversion to open	0 (0%)	0 (0%)	
Operative time (min)	120.67 (54–330)	121.31 (50–289)	0.946
Estimated blood loss (ml)	34.34 (0–200)	22.38 (0–100)	**0.026**
ICU admission	1 (1.64%)	1 (2.38%)	0.791
Length of stay (hours)	59.41 (2–1231)	17.78 (2–77)	0.095
Bile leak	0 (0%)	0 (0%)	
Intraabdominal abscess	2 (1.9%)	1 (1%)	0.793
Surgical site infection	1 (1%)	0 (0%)	0.415
Post-operative SBO	0 (0%)	0 (0%)	
Post-operative ED visit	12 (19.67%)	3 (7.14%)	0.165
Readmission (30 days)	6 (4.92%)	4 (9.52%)	0.959
Unexpected return to OR (30 days)	1 (1%)	0 (0%)	0.409
Post-operative need for ERCP	4 (6.56%)	5 (11.90%)	0.350
Blood transfusion within 30 days	0 (0%)	0 (0%)	
30-day morbidity	0 (0%)	1 (2.38%)	0.230
30-day mortality	1 (1.64%)	0 (0%)	0.409

*BMI* Body Mass Index, *ASA* American Society of Anesthesiologists Classification, *ICU* Intensive Care Unit, *SBO* Small Bowel Obstruction, *ED* Emergency Department, *ERCP* Endoscopic Retrograde Cholangiopancreatography

Bold values are statistically significant differences ( p < 0.05)

No statistically significant differences between the cohorts were detected in operative time, length of hospital stay, need for postoperative ERCP, readmissions, unexpected return to OR in 30 days, or the primary endpoint of 30-day mortality (Table 1). There were no incidences of conversion to an open procedure, bile leak, postoperative small bowel obstruction, or need for blood transfusion within 30 days across either cohort. Three intraabdominal abscesses and 1 surgical site infection were reported. A greater proportion of patients undergoing LC visited the emergency department postoperatively compared to the RC cohort, although the difference was not statistically significant. The RC group had a shorter length of stay than the LC (59.40 vs 17.78). There was a statistically significant but clinically irrelevant difference in estimated blood loss for LC compared to RC, respectively (34.34 ml vs 22.38 ml; *p* = 0.026).

Additionally, a subset analysis for emergent LC and RC cases was performed using the same set of variables. Emergent cases that were completed laparoscopically exhibited a decreased median operative time in comparison to the robotic-assisted approach (128.20 min vs 133.15 min; = 0.028) (Table 2). Although the LC cohort experiences intra-abdominal abscesses and a surgical site infection, this difference was not statistically significant (2 vs 0; *p* = 0.793 and 1 vs 0; *p* = 0.415, respectively). No statistically significant difference in estimated blood loss, ICU admission, length of stay, readmission rate, unexpected return to OR, postoperative ERCP or 30-day mortality was noted between emergent LC and emergent RC (Table 2). Only 1 patient in this cohort returned to the OR, however, it was unrelated to a bile leak or any intrabdominal complication related to the LC.

**Table 2:** Subgroup analysis of 61 emergent cholecystectomies: laparoscopic vs. Robotic

	Laparoscopic (*N* = 48)	Robotic assited (*N* = 13)	*P*-value
Age, mean (± SD)	44.5 (19–87)	60.69 (27–82)	0.191
Sex, *n* (%)			
Female	19 (18.4%)	6 (5.8%)	
Male	29 (28.2%)	7 (6.8%)	
BMI, mean (± SD)	32.26 (20.36–52.730)	30.15 (20.20–49.26)	0.273
ASA, *n* (%)			
I	3 (2.9%)	0 (0%)	
II	33 (31.1%)	4 (3.9%)	
III	12 (11.7%)	7 (6.8%)	
IV	0 (0%)	2 (1.9%)	
Conversion to open	0 (0%)	0 (0%)	
Operative time (min)	128.20 (54–330)	133.15 (50–228)	**0.028**
Estimated blood loss (ml)	35.62 (0–200)	25.76 (10–50)	0.066
ICU admission	1 (1.0%)	0 (0%)	0.791
Length of stay (hours)	67.95 (2–1231)	32.69 (3–77)	0.076
Bile leak	0 (0%)	0 (0%)	
Intraabdominal abscess	2 (1.9%)	0 (0%)	0.793
Surgical site infection	1 (1%)	0 (0%)	0.415
Post-operative SBO	0 (0%)	0 (0%)	
ED postop visit	8 (7.8%)	2 (1.9%)	0.774
Readmission (30 days)	5 (4.92%)	2 (1.9%)	0.470
Unexpected return to OR (30 days)	1 (1.0%)	0 (0%)	0.409
Post-operative need for ERCP	4 (3.9%)	4 (3.90%)	0.059
Blood transfusion within 30 days	0 (0%)	0 (0%)	
30-day morbidity	0 (0%)	0(0%)	
30-day mortality	1 (1.00%)	0 (0%)	0.409

*BMI* Body Mass Index, *ASA* American Society of Anesthesiologists Classification, *ICU* Intensive Care Unit, *SBO* Small Bowel Obstruction, *ED* Emergency Department, *ERCP* Endoscopic Retrograde Cholangiopancreatography

Bold values are statistically significant differences ( p < 0.05)

Finally, a subset analysis was performed within the RC cohort comparing the same variables between three-port and four-port RC approaches. Interestingly, three-port RC was associated with reduced operative time compared to the four-port technique (101.28 min vs 150.76 min; *p* < 0.001) in the experience of a single surgeon. While differences in emergent status of the case and need for post-operative ERCP between the four and three-port RC techniques trending towards significance, no other statistically significant differences were noted between the groups (Table 3).

**Table 3:** Outcomes of four-port and three-port robotic cholecystectomy

	Robotic 4-port (*N* = 17)	Robotic 3-port (*N* = 25)	*P*-value
Age, mean (± SD)	55.64 (27–83)	57.96 (30–82)	0.646
Sex, *n* (%)			0.183
Female	8 (47.06%)	17 (68%)	
Male	9 (52.94%)	8 (32%)	
BMI, mean (± SD)	29.67 (22.67–38.01)	29.18 (19.66–49.26)	0.785
ASA, *n* (%)			0.426
I	1 (5.89%)	0 (0%)	
II	8 (47.06%)	11 (44%)	
III	7 (41.17%)	12 (48%)	
IV	1 (5.89%)	2 (8%)	
Diagnosis			0.065
Emergent	8 (47.06%)	5 (20%)	
Planned	9 (52.94%)	20 (80%)	
Conversion to open	0 (0%)	0 (0%)	
Operative time (min)	150.76 (78–289)	101.28 (50–175)	** < 0.001**
Estimated blood loss (ml)	25 (0–100)	20.6 (0–50)	0.434
ICU admission	1 (5.89%)	0 (0%)	0.23
Length of stay (hours)	22.94 (2–76)	14.28 (2–77)	0.191
Bile leak	0 (0%)	0 (0%)	
Intraabdominal abscess	1 (1%)	0 (0%)	0.416
Surgical site infection	0 (0%)	0 (0%)	
Post-operative SBO	0 (0%)	0 (0%)	
ED postop visit	2 (11.76%)	3 (12%)	0.519
Readmission (30 days)	2 (11.76%)	2 (8%)	0.692
Unexpected return to OR (30 days)	0 (0%)	0 (0%)	
Postoperative need for ERCP	4 (23.53%)	1 (4%)	0.057
Blood transfusion within 30 days	0 (0%)	0 (0%)	
30-day morbidity	1 (5.89%)	0 (0%)	0.230
30-day mortality	0 (0%)	0 (0%)	

*BMI* Body Mass Index, *ASA* American Society of Anesthesiologists Classification, *ICU* Intensive Care Unit, *SBO*: Small Bowel Obstruction, *ED* Emergency Department, *ERCP* Endoscopic Retrograde Cholangiopancreatography

Bold values are statistically significant differences ( p < 0.05)

**Fig. 1:**
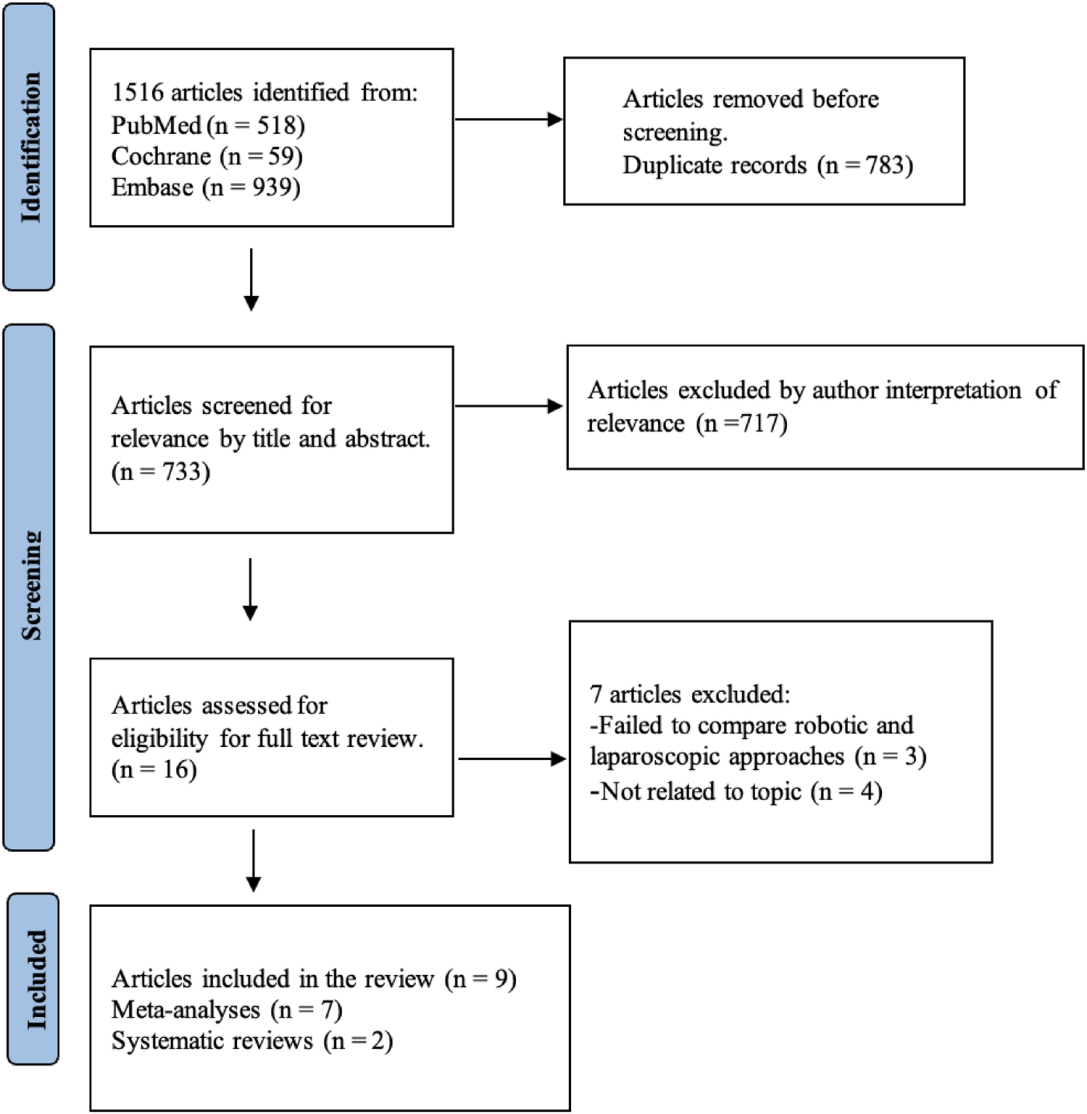
PRISMA Flow Diagram—Laparoscopic and Robotic Cholecystectomy Systematic Reviews and Meta Analyses, 2022

An umbrella systematic review of studies comparing clinical outcomes of LC and RC was conducted via comprehensive literature search of PubMed, EMBASE, and Cochrane Library databases on June 15th, 2022, which revealed 1516 articles. The umbrella systematic review as conducted based on the following inclusion criteria: only meta-analyses and/or systematic reviews published as clinical (non-experimental) studies within the last 5 years in the English language and only on human subjects who underwent LC and RC were included in our umbrella review. Exclusion criteria included non-clinical studies, not in the English language, published more than 5 years ago, and any study not classified as a meta-analysis or systematic review. Screening led to the removal of 783 articles due to duplication and 717 due to irrelevance based on title and abstract review. Sixteen articles were eligible for full-text review. Three of these articles were excluded due to a lack of comparison between LC and RC and four further excluded for irrelevance. A total of nine articles were included, consisting of seven meta-analyses [6–12] and two systematic reviews [13, 14] (Fig. 1). Altogether, 3,327,203 patients were incorporated into the umbrella review (Table 3, 4). Five out of seven meta-analyses reported significantly shorter operative times (approximately 13–17 min) for LC compared to RC [6–10], while two found no statistically significant difference [11, 12]. One study by *Huang *et al*.* reported reduced pre-operative time, defined as the time between patient entrance into the operating room and first incision, for LC (32.4 min) versus RC (53.4 min) (*p* < 0.001). Two of seven meta-analyses reported significantly reduced hospital length of stay for patients undergoing RC vs LC (0.25–0.7 days) [7, 10] with one noting a significant reduction in RC patient length of stay in non-randomized clinical trials (9). *Sun *et al. reported an increased risk of incisional hernia in patients undergoing single-site robotic cholecystectomy compared to multi-port LC (OR = 4.23), correlating with *Wang *et al.’s report of more post-operative incisional hernias in patients undergoing single-incision robotic cholecystectomy versus conventional LC patients (risk difference = 0.05, 95% confidence interval 0.02–0.07; *P* < 0.0001). Importantly, no meta-analysis or systematic review identified significant differences in intraoperative complications, rate of conversion to open surgery, estimated blood loss, or between LC and RC techniques.

**Table 4:** Summary of included peer review publications

Author	Title	Year of publication	# of patients	Study design	Approach (Laparoscopic vs Robotic)	Preoperative & operative time (in minutes)	Intraoperative complications	Conversion rate to open surgery	Estimated Blood Loss (in mL)	Postoperative Complications	Length of Hospital Stay	Readmission
*Huang *et al. [6]	Robotic cholecystectomy versus conventional laparoscopic cholecystectomy: A meta-analysis	2017	1589	Meta-analysis	LC: 921 RC: 668	Preoperative time LC: 32.4* RC: 53.4* Operative time LC: 115.3* RC: 128*	LC: 0% RC: 0%	LC: 0% RC: 0%	LC:13 mL RC: 11 mL	LC: 1.9% RC: 2.6% Incisional hernia LC & RC: 0%	LC: 2.2 days RC: 1.4 days	LC: 0.9% RC: 2.3%
*Maeso *et al. [7]	Efficacy of the Da Vinci surgical system in abdominal surgery compared with that of laparoscopy: a systematic review and meta-analysis	2010	2166	Meta-analysis	LC: 387 RC: 124	RC: 17 min longer* (range: 1–31)	Not reported	LC: 3% RC: 1%	Not reported	LC: 2% RC: 5%	RC:—0.7 days less*	Not reported
*Imsirovic *et al. [8]	Laparoscopic versus robotic cholecystectomy: A systematic review and meta-analysis of randomized controlled trials	2019	434	Meta-analysis (poster)	LC: 202 RC: 232	**SMD = 0.42** * **LC: operative time was shorter** *	Not reported	RC vs LC: RR of 1.921	RC vs LC: SMD of -0.13	Postoperative morbidity: RC vs LC: RR of 0.89	RC vs LC: SMD of 0.09	Not reported
*Han *et al. [9]	Robotic assisted versus laparoscopic cholecystectomy for benign gallbladder diseases: a systematic review and meta-analysis	2018	4004	Meta-analysis	LC: 2171 RC: 1833	MD = 13.14 * RCT—MD of 12.04 **NRCs—MD of 13.78** *	RR = 0.95 RCT – RR of 0.87 NRCs – RR of 1.11	RR = 0.53 RCT – RR of 0.50 NRCs – RR of 1.00	MD = -0.95 NRCs- MD of- 0.92 RCT – MD of—1.07	RR = 0.78 RCT – RR of 1.76 NRCs – RR of 0.67 Incisional hernia RR = 3.22 RCT- RR of 7 NRCs – RR of 3.06	MD = -0.20* RCT- 0.05 **NRCs—− 0.27** *	RAC vs LC: RR of 1.21
*Aleuy *et al. [10]	Robotic Versus Laparoscopic Cholecystectomy: A Meta Analysis	2022	3,196,661	Meta-analysis (poster)	LC: 3,148,234 RC: 43,202	**RC vs LC:MD of 19.686** *	RC vs LC: MD of 1.174	RC vs LC: MD of 0.457	RC vs LC: MD of -6.143	RC vs LC: MD of 1.174	RC vs LC: MD of − 0.25 days *	Not reported
*Sun *et al. [11]	Single-incision robotic cholecystectomy versus single-incision laparoscopic cholecystectomy: A systematic review and meta-analysis	2018	633	Meta-analysis	SILC: 328 SIRC: 305	SIRC vs SILC: MD of 17.32	SIRC vs SILC: OR of 0.48	SIRC vs SILC: OR of 0.52	Not reported	SIRC vs SILC: OR of 0.62	SIRC vs SILC: MD of—0.01	SIRC vs SILC: OR of 0.70
*Sun *et al. [12]	Single-site robotic cholecystectomy versus multi-port laparoscopic cholecystectomy: A systematic review and meta-analysis	2018	1657	Meta-analysis	MLC: 882 SSRC: 775	SSRC vs MLC: MD of -3.06	Not reported	SSRC vs MLC: OR of 1.3	SSRC vs MLC: OR of = 0. 38	SSRC vs MLC: OR of 1.11 Wound infection: OR of 1.92 Incisional hernia: OR of 4.23*	SSRC vs MLC: MD of -0.02	Not reported
*Shenoy *et al. [13]	Intraoperative and postoperative outcomes of robot-assisted cholecystectomy: a systematic review	2021	112,231	Systematic Review	LC: 111,507 RC: 724	RCT: RC - 61–98 min LC – 48–87 min PMI: RC – 80–185 min LC – 60.2–160 min	RCT: RC- 0–40% LC- 0–46.7%	RCT: RC: 0–6.7% LC: 0–10%	Not reported	SSI (RCT): RC: 2.4–6% LC: 0–3.3%	RCT: RC – 16.7 h–1.9 days LC- 13.9 h–3.1 days PM: RC- 0.1–4.92 LC – 0.14–5.7	Not reported
*Wang *et al. [14]	Laparoscopic surgery and robotic surgery for single-incision cholecystectomy: an updated systematic review	2021	7828	Systematic Review	SILC: 6,234 SIRC: 1,544 Not specified: 50	SILC: 68.2 min SIRC: 88.8 min	SILC: SIRC:	SILC: 0.75% SIRC: 1.5%	SILC: 12.1 SIRC: 14.8	SILC: 6.2% SIRC: 4.1% SIC vs CLC: 5.6% vs 4.5% Incisional hernia: SILC: 1.4% SIRC: 4.8% SIRC vs CLC = 6.4% vs 1.3%*	SILC: 2.8 days SIRC: 1.9 days	Not reported

Bold* = statistically significant

*mL* milliliters, *SMD* standard mean difference, *MD* mean difference, *RAC* robotic-assisted cholecystectomy, *NRCs* non-randomized clinical trial, *RCT* randomized clinical trial, *PM* propensity matched, *SSI* surgical site infection, *SILC* single incision laparoscopic cholecystectomy, *MILC* multiple incision laparoscopic cholecystectomy, *SIRC* single incision robotic cholecystectomy, *MLC* multi port robotic cholecystectomy, *SSRC* single site robotic cholecystectomy, *CLC* conventional laparoscopic cholecystectomy

## Discussion

This study compared differences in outcomes between LC and RC by a single surgeon at an academic medical center with a young robotic surgery program. Patients undergoing RC were older, exhibited lower BMI and a higher proportion underwent planned rather than emergent cholecystectomy. In those undergoing RC, the three-port approach was associated with reduced operative time compared to the four-port approach. Importantly, no differences were detected in length of hospital stay, need for postoperative ERCP, readmissions, re-operation, or 30-day mortality between LC and RC. This series supports the observation that RC can be non-inferior to LC in the hands of surgeons comfortable with both techniques. Either approach by a single surgeon was feasible, effective, and safe in an urban academic medical center and minimal and comparable complications were noted in both minimally invasive approaches.

Interestingly, the robotic approach did not increase operative time compared to the laparoscopic approach even with heavy emphasis on resident training across both modalities. On the contrary, the robotic approach contributed to a reduction in hospital length of stay. This finding may vary with individual surgeon and resident competency in the robotic approach. Of note, residents in the cases analyzed were required to complete a formal academic robotic surgery curriculum prior to participating. It should also be noted that a higher proportion of patients undergoing LC required emergent intervention, which may indicate a more severe disease process with increased inflammation and thus longer operative times. This finding may also be due to a lack of availability of robotic platforms or adequately trained personnel support which is also highly variable across institutions.

Many variations to surgical techniques can affect clinical outcomes, including in RC. The number of ports utilized for RC and their placement are constantly being revised and studied for clinical impact [15–17]. In theory, the structure of the robotic wrist optimizes retraction angles, making three-port RC safe and efficient [18]. In this series, three-port RC was associated with significant reduction in operative time compared to four-port RC. This observation may be a reflection of surgeon judgement that more severe disease and inflammation necessitate a fourth robotic arm to aid in retraction as opposed to a stationary internal retraction stitch between the gallbladder fundus right upper quadrant abdominal wall as is customary in the three-port technique. Nevertheless, identification and documentation of the critical view of safety by an experienced surgeon, regardless of the minimally invasive modality or number of ports utilized, is the most crucial factor in performing a safe cholecystectomy [19].

The results of the umbrella systematic review reiterate the feasibility and safety of RC compared to LC. No difference in the rate of conversion of open surgery or readmission was identified, in line with our single surgeon study. Our findings also demonstrate a reduction in hospital length of stay for RC patients in concordance with several larger analyses. While our series did not detect a difference in operative time, several meta-analyses reported significantly shorter operative times for LC compared to RC [6–10]. Several factors may account for this, including surgeon skill and comfort with each approach and institutional experience and capability with the more recently developed robotic platform. In fact, *Huang *et al*.*’s finding of reduced pre-operative time in LC versus RC alludes to likely contributions of institutional and support staff learning curves to increased operative time in robotic versus laparoscopic approaches. As surgeons and surgical teams continue to develop mastery across minimally invasive techniques, the learning curve may flatten in robotic surgery [20, 21]. Furthermore, regular incorporation of residents trained in the use of the robotic platform did not cause any delays or increased complications in RC compared to the LC approach on which they are more traditionally trained.

Many studies have explored the advantages of employing robotic systems, highlighting their ability to provide enhanced visualization of critical anatomical structures and potentially lead to easier and safer cystic artery and duct dissection, ligation, and transection [11, 12]. The robotic approach can be associated with more precision, improved depth perception, and ergonomic advantage, in turn decreasing surgeon stress and physical burden [9]. It can better equip the surgeon to successfully deal with complicated cases in such a way that the risk for conversion is significantly reduced [6], especially given that cholecystectomy in acute and chronic cholecystitis have been associated with an increased rate of open conversion when attempted laparoscopically [14]. Furthermore, single incision RC has been reported to provide a more magnified, stable, and high-definition 3D image with tremor suppression compared to single incision LC, which positively affect patient outcomes and decrease injury to the biliary tract and other systems [11].

While single incision RC was associated with a higher risk of incisional hernias in the umbrella systematic review, caution is warranted when interpreting these results due to the marked variance in fascial closure techniques, follow up times, and the innate fascial weakness of the periumbilical region [14]. Given that an increased rate of incisional hernias has not been consistently reported, more research is needed to identify any true association.

This study has several important limitations, including its single surgeon scope. Surgeon volume and experience are among the most predictive factors of patient outcomes across multiple surgical specialties [22–25], making it difficult for the results of this series to apply to a variety of surgeon experience. While larger samples were analyzed in the umbrella systematic review, high heterogeneity between studies and lack of cohesion among reporting variables and definitions may diminish its power. Additionally, the study’s retrospective nature along with the inherent patient factors persuading surgeons to use one surgical approach versus another make it prone to selection bias. The most effective future studies should include prospective and blinded randomized controlled trial methodology with the addition of cost analysis for both elective and emergent indications.

Minimally invasive surgery, whether laparoscopic or robotic assisted, has become an essential part of current surgical practice. The adoption of robotic surgery is often associated with a learning curve in all respects, from the technique itself to billing and reimbursement. In the future, when healthy competition inevitably builds within the industry, the benefits of a more natural and manageable learning curve may outweigh the disadvantages. As the use of robotic surgery becomes more prevalent, its access will increase, and the limitations introduced by the cost of the platform and its maintenance will decrease. Eventually, as we move towards the ultimate goal of facilitating the most optimal hospital and patient outcomes, robotic general surgery will continue to become the norm, not the exception.

An important limitation of our study is the fact that the seven studies that met inclusion criteria for our umbrella systematic review are heterogeneous in terms of their patient population, and techniques. Unfortunately, it is one of the inherent weaknesses of our study and design. However, given the fact that only seven studies met the rigorous inclusion criteria that we established, we considered them appropriate for an umbrella systematic review due to the fact that very few publications add to the literature in this specific instance and for this focused topic of interest, which is an objective comparison between LC and RC based on clinical outcomes data.

Future research should focus on analysis of large multi-institutional databases, systematic reviews, meta-analyses, and prospective randomized controlled trials dealing with the adoption of robotic surgery for elective and emergency general surgery in community and academic center environments. However, an equally important and relevant issue is the responsible adoption of robotic surgery with a cost-reducing and financially feasible mindset in general surgery as the speciality with the fastest adoption of the robotic technology [26].

## Conclusion

Robotic cholecystectomy is feasible and safe at a young robotic surgery program in an academic center setting and comparable to laparoscopic cholecystectomy clinical outcomes.

## Supplementary Information

Below is the link to the electronic supplementary material.

Supplementary file1 (XLSX 25 KB)

## Data Availability

The data that support the findings of this study are not openly available and are available from the corresponding author upon reasonable request.
